# Genomic prediction using imputed whole-genome sequence data in Holstein Friesian cattle

**DOI:** 10.1186/s12711-015-0149-x

**Published:** 2015-09-17

**Authors:** Rianne van Binsbergen, Mario P. L. Calus, Marco C. A. M. Bink, Fred A. van Eeuwijk, Chris Schrooten, Roel F. Veerkamp

**Affiliations:** Animal Breeding and Genomics Centre, Wageningen UR Livestock Research, PO Box 338, 6700 AH Wageningen, The Netherlands; Biometris, Wageningen University and Research Centre, PO Box 16, 6700 AA Wageningen, The Netherlands; CRV, Arnhem, The Netherlands

## Abstract

**Background:**

In contrast to currently used single nucleotide polymorphism (SNP) panels, the use of whole-genome sequence data is expected to enable the direct estimation of the effects of causal mutations on a given trait. This could lead to higher reliabilities of genomic predictions compared to those based on SNP genotypes. Also, at each generation of selection, recombination events between a SNP and a mutation can cause decay in reliability of genomic predictions based on markers rather than on the causal variants. Our objective was to investigate the use of imputed whole-genome sequence genotypes versus high-density SNP genotypes on (the persistency of) the reliability of genomic predictions using real cattle data.

**Methods:**

Highly accurate phenotypes based on daughter performance and Illumina BovineHD Beadchip genotypes were available for 5503 Holstein Friesian bulls. The BovineHD genotypes (631,428 SNPs) of each bull were used to impute whole-genome sequence genotypes (12,590,056 SNPs) using the Beagle software. Imputation was done using a multi-breed reference panel of 429 sequenced individuals. Genomic estimated breeding values for three traits were predicted using a Bayesian stochastic search variable selection (BSSVS) model and a genome-enabled best linear unbiased prediction model (GBLUP). Reliabilities of predictions were based on 2087 validation bulls, while the other 3416 bulls were used for training.

**Results:**

Prediction reliabilities ranged from 0.37 to 0.52. BSSVS performed better than GBLUP in all cases. Reliabilities of genomic predictions were slightly lower with imputed sequence data than with BovineHD chip data. Also, the reliabilities tended to be lower for both sequence data and BovineHD chip data when relationships between training animals were low. No increase in persistency of prediction reliability using imputed sequence data was observed.

**Conclusions:**

Compared to BovineHD genotype data, using imputed sequence data for genomic prediction produced no advantage. To investigate the putative advantage of genomic prediction using (imputed) sequence data, a training set with a larger number of individuals that are distantly related to each other and genomic prediction models that incorporate biological information on the SNPs or that apply stricter SNP pre-selection should be considered.

**Electronic supplementary material:**

The online version of this article (doi:10.1186/s12711-015-0149-x) contains supplementary material, which is available to authorized users.

## Introduction

Genomic selection is increasingly applied in breeding programs for livestock and plant species, e.g. [1–4]. Genomic selection relies on the prediction of genomic estimated breeding values (GEBV) of individuals or lines using marker genotype information only, by applying genomic prediction models that are based on training individuals that have both phenotypic and genotypic data. In most breeding programs, single nucleotide polymorphism (SNP) marker panels are used. With SNP panels, the level of linkage disequilibrium (LD) between SNPs and the actual causal variant (e.g. SNP, insertion, deletion, etc.) influences the reliability of genomic prediction. In this paper, these causal variants will be considered as quantitative trait loci (QTL). At each generation of selection, recombination events between a SNP and the QTL can cause a decay in the reliability of genomic predictions [5]. Typically, a decrease in reliability of GEBV prediction in cattle with 50k SNP genotypes has been observed when the additive-genetic relationships between training animals and validation animals decrease [6, 7]. Moreover, this decay in reliability was greater when the size of the training set was smaller [6]. This decay could become a problem for dairy cattle since sons from young bulls that are selected on their GEBV without daughter information are now entering breeding programs. These sons’ GEBV are estimated based on a training set of progeny-tested bulls that are two generations older (i.e. their grand sires) and therefore the reliability of their genomic breeding values will be lower compared with those of the previous generation.

On average, increasing the number of SNPs in a panel increases the level of LD between a SNP and a QTL and this should be beneficial for genomic prediction. Studies using real data, have shown that genomic prediction using an array with approximately 777,000 (imputed) SNPs resulted in a small gain in genomic prediction reliability compared to an array with approximately 50,000 SNPs [8–10]. However, even with 777,000 SNPs, predictions still depend on LD between SNPs and QTL. In contrast to the SNP panels currently used, whole-genome sequence data are expected to include the causal mutations that underlie QTL [11], which means that predictions should no longer depend on LD between SNPs and QTL. Inclusion of the causal mutations allows the effect of the QTL on a given trait to be estimated directly, which should increase the reliability of genomic predictions compared to using SNP genotypes, as well as the persistency of the reliability of predictions across generations and even across breeds [11–13].

However, identifying the QTL and obtaining a higher persistency of reliabilities of genomic predictions over generations probably requires a large training set of thousands of sequenced individuals. Without a large number of training individuals, QTL effects might be estimated with too much error and thus, there will be little advantage of using sequence data [14]. Sequencing many individuals is still too expensive but instead imputed sequence data can be used, especially since many animals that are genotyped using SNP panels are available in livestock populations.

The 1000 bull genome project [15] aims at sequencing a number of key ancestor bulls in the beef and dairy cattle population at medium coverage. These sequenced animals can be used as reference animals to impute other animals that are genotyped with 50k or 777k SNP panels to the whole-genome sequence level. A reliability of 0.83 was obtained for imputation from 777k SNP panels to sequence data with a reference set of 91 Holstein Friesian animals with whole-genome sequence data [16]. Moreover, adding individuals of other breeds in a relatively large reference set will further increase imputation accuracy. In particular, it was reported that low MAF (minor allele frequency) variants that segregate in other breeds can benefit from combining different breeds together [17, 18]. Therefore, imputation to sequence data using SNP genotypes is an attractive and cost-effective approach to obtain a large training set of sequenced individuals, and to investigate the benefit of using sequence data for relevant populations.

Many methods are available for genomic prediction, most of which are based on linear regression (see [19] for review). These methods can differ in the underlying assumptions about the distribution of SNP effects. With a genome-enabled best linear unbiased prediction (GBLUP) model it is assumed that the a priori variance of SNP effects is equal, so a large number of SNPs, each with a small effect, are fitted in the model (infinitesimal model). Consequently, it is expected that GBLUP does not take full advantage of sequence data, since it will allocate the same variance to SNPs without effect and to those that are causal, although only a very small proportion of the SNPs is expected to be causal. Alternatively, methods such as BayesB [20], BayesC [21] and Bayesian stochastic search variable selection (BSSVS) [22, 23] assume that the a priori variance of the effects of many SNPs is very small or zero, while it is large for only a few SNPs. Because of this mixture of the prior distributions of SNP effects, these methods could benefit from sequence data. Simulation studies using bovine sequence data confirmed this expectation, e.g. [11–13]. However, Ober et al. [24] concluded that predictions from BayesB were not better than predictions from a method equivalent to GBLUP when using real sequence genotypes of *Drosophila melanogaster*, although the size of the training set size (~120 observations) was relatively small. Moreover, the advantage of Bayesian methods over GBLUP was shown to be greatly influenced by the size and distribution of the simulated QTL effects [11–13].

Since the use of whole-genome sequence data for genomic prediction in livestock populations, and its impact on the reliability of genomic prediction and persistency across generations have been mainly studied with simulated data, the objective of this study was to investigate (the persistency of) the reliability of genomic predictions based on imputed whole-genome sequence genotypes versus 777k SNP genotypes for real dairy cattle data.

## Methods

### Phenotypes

De-regressed proofs and associated weights (effective daughter contributions, EDC) were available for somatic cell score (SCS), interval between first and last insemination (IFL), and protein yield (PY) for 5503 Holstein Friesian bulls provided by CRV (Arnhem, the Netherlands). De-regressed proofs (DRP) were calculated according to VanRaden et al. [25]:

$$ \mathrm{D}\mathrm{R}\mathrm{P}=\mathrm{P}\mathrm{A}+\left(\mathrm{E}\mathrm{B}\mathrm{V}-\mathrm{P}\mathrm{A}\right)\ast \left(\frac{{\mathrm{EDC}}_{\mathrm{EBV}}}{{\mathrm{EDC}}_{\mathrm{prog}}}\right), $$

where EBV is the estimated breeding value of a bull for a trait available from the national evaluations, and PA is the parent average of the bull for that trait. Effective daughter contribution, EDC_EBV_, represents the effective number of daughters with phenotypes that contributed to the estimated breeding value of a bull [26] and was calculated according to VanRaden and Wiggans [27] as *α* ∗ REL_EBV_/(1 − REL_EBV_), where REL_EBV_ is the published reliability for EBV and *α* = (4 − *h*^2^)/*h*^2^, where *h*^*2*^ is the heritability of the trait. EDC_prog_ = EDC_EBV_ − EDC_PA_, where EDC_PA_ = *α*REL_PA_/(1 − REL_PA_) and REL_PA_ = (REL_sire_ + REL_dam_)/4 [27]. As the number of daughters with phenotypic information for a trait increases, the reliability of the EBV of a bull and EDC_EBV_ increase. The average EDC_EBV_ (and its range) for animals in the training set was equal to 266 (24–971) for SCS, 643 (47–4851) for IFL, and 245 (24–693) for PY. The pedigree information for the 5503 bulls in this study included 39,917 animals.

### Genotypes

In total, 551 bulls were genotyped with the Illumina BovineHD BeadChip (Illumina Inc., San Diego, CA) and the other 4952 bulls were genotyped with a 50k SNP panel and imputed to BovineHD (734,403 SNPs). Imputation from the 50k to the BovineHD SNP panel was performed with Beagle 3.3.0 [28, 29], using a reference set of 1333 animals genotyped with the BovineHD SNP panel. For this first step, the error rate of imputation was low [30]. For each bull, BovineHD genotypes were subsequently imputed to whole-genome sequence genotypes with Beagle version 4 [31]. The following default parameter settings in Beagle were used: five iterations for initial burn-in, five iterations for phasing, and five iterations for imputation. Imputation was performed for sliding windows of 24,000 SNPs in the sequence data, with an overlap of 3000 SNPs between sliding windows. No pedigree information was used in the imputation procedure. The sex chromosomes were excluded.

Whole-genome sequence data (28,336,153 SNPs) of 429 animals that were provided by the 1000 bull genomes project (Run 3.0) were used as reference data for imputation. All these animals, except two, were males and originated from 15 dairy and beef breeds (1 to 121 animals per breed), among which there were four major breeds, with 121 Holstein, 87 Simmental, 54 Angus, and 43 Brown Swiss animals. Each animal was sequenced with the Illumina HiSeq System (Illumina Inc., San Diego, CA). Alignment, variant calling, and quality controls were as described by Daetwyler et al. [15]. The average number of sequence genotypes was equal to 9.6 per animal and ranged from 3.0 to 44.5. To assess the accuracy of genotype calling, concordance with BovineHD genotypes was calculated as the proportion of identical genotypes between the BovineHD and sequence data and ranged from 67.5 to 99.9 % (on average 94.8 %) for the 303 animals with BovineHD genotypes. After correcting sequence genotypes with Beagle, average concordance increased to 98.3 % (range: 74.1–99.9 %). Note that most animals in this whole-genome sequence dataset were only used as reference animals for imputation and not for genomic prediction, except for 57 bulls that had genotypes in both datasets.

After imputation, non-informative SNPs were removed from the dataset, i.e. SNPs with less than two alleles, SNPs with a minor allele frequency lower than 0.005 and SNPs with an estimated imputation reliability lower than 0.05 (only for the imputed sequence data). Imputation reliability was predicted by Beagle software as the estimated squared correlation between the estimated allele dosage (0∗P(AA) + 1∗P(AB) + 2∗P(BB)) and the true allele dosage (estimated from posterior genotype probabilities) [32]. In general, the imputation reliability predicted by Beagle gives a good indication of the true reliability for imputation from BovineHD to sequence data [16]. The thresholds for these selection criteria were chosen so that monomorphic SNPs and SNPs that are likely to be imputed incorrectly were removed.

To evaluate the effect of imputation on genomic prediction, a third genotype panel (ImputedHD) was generated by randomly selecting SNPs from the imputed sequence data. The number of selected SNPs per chromosome was the same as for the BovineHD genotype dataset, and did not include SNPs that were in the BovineHD genotype dataset.

### Genomic prediction

GEBV for the three traits were predicted based on two sets of genotypes: the original BovineHD genotypes and imputed whole-genome sequence genotypes. In both cases, the most likely genotypes were used for prediction. Genomic prediction was performed using two types of linear regression models: GBLUP and BSSVS.

#### GBLUP

The GBLUP model was:

$$ \mathbf{y}=\mathbf{1}\mu +\mathbf{Zg}+\mathbf{e}, $$

where **y** is the vector of de-regressed proofs of all individuals, *μ* is the overall mean, **1** is a vector of ones, **Z** is an incidence matrix that links records to bulls, **g** is a matrix of the genomic breeding values of all individuals, and **e** contains the random residuals. Genomic breeding values were assumed to be distributed as **g**|**GRM**, *σ*_*g*_^2^ ~ *N*(0, **GRM***σ*_*g*_^2^), where **GRM** is the genomic relationship matrix, and *σ*_*g*_^2^ is the additive genetic variance picked up by the markers. Diagonal and off-diagonal values of the **GRM** were calculated following Yang et al. [33] as:

$$ {G}_{jk}=\frac{1}{N}{\displaystyle \sum i}{G}_{ijk}=\frac{1}{N}{\displaystyle \sum i}\frac{\left({x}_{ij}-2{p}_i\right)\left({x}_{ik}-2{p}_i\right)}{2{p}_i\left(1-{p}_i\right)}, $$

where *G*_*ijk*_ is the estimated relationship between individuals *j* and *k* at locus *i*, and *N* is the number of SNPs. The SNP genotypes (*x*_*i*_) were coded as 0, 1 or 2, and *p*_*i*_ is the allele frequency of the allele for which the homozygote genotype was coded as 2. Residual effects were assumed to be distributed as **e**|**D**, *σ*_*e*_^2^ ~ *N*(0, **D***σ*_*e*_^2^), where **D** is a diagonal matrix containing 1/EDC_EBV_ on the diagonals, and *σ*_*e*_^2^ is the residual variance.

After calculation of the genomic relationship matrix, the GBLUP model was fitted using the ASReml 4 software [34]. ASReml software was used to estimate variance components (restricted maximum likelihood estimation, REML), with BLUP of the random effects as ‘byproducts’. Therefore, it might be more appropriate to call this method GREML. However, since our main objective was to predict genomic values; we used the terminology GBLUP.

#### BSSVS

The BSSVS model [23] was:

$$ \mathbf{y}=\mathbf{1}\mu +\mathbf{Z} \mathbf{u}+\mathbf{X}\boldsymbol{\upalpha } +\mathbf{e}, $$

where **u** is a vector that contains the polygenic effects of all bulls (**u**|**A**, *σ*_*u*_^2^ ~ *N*(0, **A***σ*_*u*_^2^), where **A** is the numerator relationship matrix derived from the pedigree), **X** is a matrix that contains the allele dosage (0, 1, or 2) for all SNPs (rows) for all bulls (columns), **α** is a vector that contains the (random) allele substitution effects for all SNPs. The prior for *μ* was a constant and both *σ*_*u*_^2^ and *σ*_*e*_^2^ had a flat, uninformative prior distribution.

An important aspect of the BSSVS is that the prior distribution for each allele substitution effects for each locus *j* (*α*_*j*_) depends on the variance for the allele substitution effects (*σ*_*α*_^2^) and the QTL indicator *I*_*j*_, which is sampled for each locus *j* and takes the value 0 (1) if the SNP was included in the model with a small (large) effect:

$$ {\alpha}_j\Big|{I}_j,{\sigma}_{\alpha}^2=\left\{\begin{array}{c}\hfill \sim N\left(0,\frac{\sigma_{\alpha}^2}{100}\right)\  when\ {I}_j=0\hfill \\ {}\hfill \sim N\left(0,{\sigma}_{\alpha}^2\right)\  when\ {I}_j=1.\hfill \end{array}\right. $$

The prior distribution for *I*_*j*_ was: *p*(*I*_*j*_) = Bernoulli(1 − *π*). For both the BovineHD and the imputed sequence datasets, the same number of SNPs (885) was assumed to have a large effect, therefore *π* was assigned a value equal to (*n*_*total*_ − 885)/*n*_*total*_, where *n*_*total*_ is the total number of SNP effects. The prior distribution for *σ*_*α*_^2^ was: *p*(*σ*_*α*_^2^) = *χ*^− 2^(*v*_*a*_, *S*_*a*_^2^), with *v*_*a*_ = 4.2 degrees of freedom [20, 21], and scale parameter ${S}_a^2=\frac{{\tilde{\sigma}}_{\alpha}^{2}\left({v}_{a}-2\right)}{v_a}$, where ${\tilde{\sigma}}_{\alpha}^2=\left(\frac{100}{100+\pi \left(1-100\right)}\right)\frac{\sigma_g^2}{n_{total}}$ [19].

The conditional posterior densities of the BSSVS model are described in Additional file 1 (See Additional file 1). The additive genetic variance (σ_*g*_^2^) was estimated as the sum of the posterior mean variances explained by the SNPs (σ_SNP_^2^) and estimated variance of the polygenic effect included in the BSSVS model (σ_*u*_^2^), where ${\upsigma}_{\mathrm{SNP}}^2={\displaystyle \sum j=1{n}_{total}}{\alpha}_j^2$. The BSSVS model was implemented using Gibbs sampling, using right-hand-side updating as described in [23], and was run in three chains per trait of 80,000 cycles, with the first 10,000 cycles disregarded for burn-in. Burn-in length was chosen based on a preliminary study using a similar dataset [35]. Convergence of the BSSVS model was monitored by plotting the total SNP variance for each cycle of the Gibbs sampler (See Additional file 2: Figure S1). For each trait, the results (variances and BLUPs) of three chains were combined.

#### Pedigree BLUP

For comparison, BLUP based on pedigree information only was also performed. Following the notation above, the model was:

$$ \mathbf{y}=\mathbf{1}\mu +\mathbf{Z}\mathbf{u}+\mathbf{e}. $$

Similar to GBLUP, the BLUP model was applied using ASReml 4 software [34].

### Prediction reliability

The reliability of genomic prediction was evaluated by assigning all 5503 bulls to either the training or validation set based on year of birth, according to the protocol used to validate genomic prediction in practice. Bulls born before 2001 (3416 bulls) were assigned to the training set and bulls born between 2001 and 2008 (2087 bulls) to the validation set. The validation animals were split into smaller subgroups (see below) to ensure that the number of animals in these subgroups was sufficient, and a relatively large number of validation animals were chosen. Reliability of genomic prediction was calculated for the validation animals as the squared correlation between de-regressed proof and the EBV for the different traits. Furthermore, the regression coefficient of the DRP on the EBV was calculated to evaluate the bias of predictions. A regression coefficient of 1 indicates no bias.

Persistency of the reliability of genomic prediction across generations was evaluated by splitting the validation bulls into three non-overlapping groups based on the presence of close relatives in the training set. The first group consisted of 1643 bulls with their sire and maternal grandsire in the training set (SMGS); the second group consisted of 113 bulls with their sire in the training set, but no maternal grandsire (SIRE); and the third group consisted of 329 bulls with no sire in the training set, but had one or both grandsires in the training set (GS). Two animals had no sire and no grandsires in the training set, and therefore were excluded from these analyses.

## Results

### Descriptive results

**Fig. 1:**
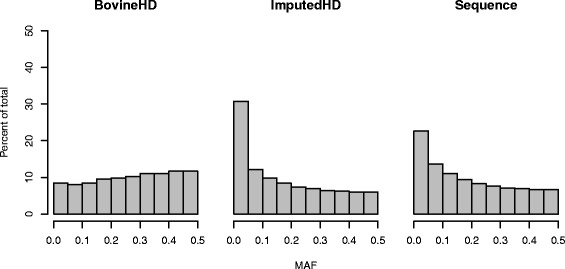
Distribution of minor allelic frequencies (MAF) among 5503 individuals for different genotype panels

After editing SNPs for MAF and imputation reliability, the final BovineHD and ImputedHD genotype dataset consisted of 631,428 SNPs and the imputed sequence genotype dataset of 12,590,056 SNPs. In the final datasets, the average minor allele frequency (MAF) was equal to 0.27 with a median of 0.28 for the BovineHD dataset, 0.17 with a median of 0.13 for the ImputedHD dataset and 0.19 with a median of 0.16 for the imputed sequence dataset. The distribution of SNPs across the different classes of MAF is in Fig. 1. Imputation reliability estimated by Beagle was on average 0.77 and ranged from 0.05 to 1.00, with a median of 0.89. Across prediction methods, the additive genetic variance (sum of polygenic and total SNP variance for BSSVS; SNP variance for GBLUP; polygenic variance for BLUP) ranged from 17.0 to 20.2 for SCS, from 15.9 to 19.6 for IFL and from 285.4 to 341.1 for PY (Table 1). As expected for de-regressed proofs, estimates of residual variance were very small, and therefore heritability estimates for all traits were close to 1 (Table 1).

**Table 1:** Estimates of genetic parameters

Trait	Genotype data	Method	*σ*_*g*_^2^	*h* ^2^	*b* ^(a)^	*r* ^2^ ^(b)^
SCS	Pedigree	BLUP	20.22	0.97	1.00	0.33
	BovineHD	GBLUP	16.97	0.90	0.96	0.52
	BovineHD	BSSVS	18.55	0.95	0.99	0.52
	ImputedHD	GBLUP	17.41	0.93	1.00	0.50
	ImputedHD	BSSVS	18.37	0.98	1.05	0.51
	Sequence	GBLUP	17.09	0.93	1.03	0.49
	Sequence	BSSVS	18.82	0.98	1.04	0.50
IFL	Pedigree	BLUP	19.60	1.00	0.92	0.27
	BovineHD	GBLUP	15.90	0.94	0.83	0.39
	BovineHD	BSSVS	18.01	0.99	0.92	0.40
	ImputedHD	GBLUP	16.29	0.95	0.86	0.37
	ImputedHD	BSSVS	17.20	1.00	0.97	0.39
	Sequence	GBLUP	16.13	0.96	0.88	0.37
	Sequence	BSSVS	17.71	1.00	0.95	0.39
PY	Pedigree	BLUP	341.05	1.00	0.82	0.26
	BovineHD	GBLUP	295.05	0.94	0.86	0.47
	BovineHD	BSSVS	306.53	0.99	0.89	0.48
	ImputedHD	GBLUP	307.33	0.97	0.89	0.44
	ImputedHD	BSSVS	285.36	1.00	0.95	0.45
	Sequence	GBLUP	300.68	0.98	0.92	0.44
	Sequence	BSSVS	293.73	1.00	0.95	0.45

Estimates of additive genetic variance (*σ*_*g*_^2^), heritability (*h* ^2^), regression coefficient (*b*), and prediction reliability (*r* ^2^) for somatic cell score (*SCS*), interval between first and last insemination (*IFL*), and protein yield (*PY*) using four types of data and two prediction methods. ^a^Standard error of the regression coefficient ranged from 0.02 to 0.03; ^b^standard error of the prediction reliability was 0.02

### Prediction reliabilities

Prediction reliabilities ranged from 0.26 to 0.52 on average (Table 1). Overall, reliabilities were highest for SCS and lowest for IFL, except for pedigree-based BLUP, for which PY had the lowest reliability. For all traits, pedigree-based BLUP gave the lowest reliabilities and GBLUP performed less well than BSSVS. For both genomic prediction methods, reliabilities were highest when the BovineHD genotype data was used. Correlations between predicted breeding values using the different datasets and different genomic prediction methods were high and ranged from 0.95 to 1.00 (See Additional file 3: Table S1). For SCS, the coefficients of regression of the original phenotypes on the predicted breeding values were close to 1.00 (ranged from 0.96 to 1.05; Table 1). For IFL and PY, a slight overestimation of the breeding values was observed, since the regression coefficients ranged from 0.82 to 0.97 (Table 1). Using imputed sequence data, the overestimation for IFL and PY was less than when using BovineHD data, i.e. the regression coefficients were closer to 1.00. Plots of the de-regressed proofs versus the GEBV (for the two methods using the three types of data) for the 2087 validation animals and three traits are in Figures S2, S3 and S4 (See Additional file 2: Figures S2, S3 and S4).

To evaluate the reliability of genomic predictions across generations, the validation bulls were divided into groups based on the presence of (grand)parents in the training set: sire and maternal grandsire (SMGS); only sire (SIRE); no sire, but one or two grandsires (GS). As expected, in most cases, the SMGS group had the highest prediction reliability and the GS group the lowest (Table 2). Overall, across those groups, the largest decay in prediction reliability was found for IFL. Moreover, for IFL, the decay in prediction reliability was larger with both ImputedHD data and imputed sequence data (in both cases, the decay was equal to −35 % for GS compared to SMGS) than with BovineHD data (−25 % for GS compared to SMGS). For SCS and PY, this difference was much smaller (Table 2). Overall, there was no clear benefit of using sequence data on the persistency of reliability across generations, even when BSSVS was used.

**Table 2:** Estimated prediction reliability per pedigree group

Trait	Genotype data	Method	SMGS^a^	SIRE^b^ (% of SMGS)	GS^c^ (% of SMGS)
SCS	Pedigree	BLUP	0.35	0.33 (94 %)	0.23 (67 %)
	BovineHD	GBLUP	0.53	0.50 (95 %)	0.45 (85 %)
	BovineHD	BSSVS	0.53	0.51 (95 %)	0.46 (86 %)
	ImputedHD	GBLUP	0.51	0.52 (101 %)	0.42 (83 %)
	ImputedHD	BSSVS	0.52	0.52 (102 %)	0.44 (85 %)
	Sequence	GBLUP	0.50	0.53 (104 %)	0.43 (85 %)
	Sequence	BSSVS	0.51	0.53 (103 %)	0.44 (87 %)
IFL	Pedigree	BLUP	0.29	0.16 (55 %)	0.15 (51 %)
	BovineHD	GBLUP	0.40	0.34 (85 %)	0.30 (75 %)
	BovineHD	BSSVS	0.42	0.34 (80 %)	0.31 (74 %)
	ImputedHD	GBLUP	0.39	0.32 (81 %)	0.25 (65 %)
	ImputedHD	BSSVS	0.41	0.31 (75 %)	0.27 (65 %)
	Sequence	GBLUP	0.39	0.32 (83 %)	0.25 (65 %)
	Sequence	BSSVS	0.41	0.32 (78 %)	0.27 (65 %)
PY	Pedigree	BLUP	0.30	0.30 (101 %)	0.24 (81 %)
	BovineHD	GBLUP	0.48	0.48 (100 %)	0.45 (95 %)
	BovineHD	BSSVS	0.49	0.49 (101 %)	0.45 (91 %)
	ImputedHD	GBLUP	0.45	0.43 (96 %)	0.41 (91 %)
	ImputedHD	BSSVS	0.47	0.47 (100 %)	0.41 (88 %)
	Sequence	GBLUP	0.45	0.45 (99 %)	0.42 (93 %)
	Sequence	BSSVS	0.46	0.45 (98 %)	0.42 (90 %)

Estimates of prediction reliability for somatic cell score (*SCS*), interval between first and last insemination (*IFL*), and protein yield (*PY*). Validation animals were divided based on the presence of relatives in the training set: sire and maternal grandsire (*SMGS*); only sire (*SIRE*); no sire, but one or two grandsires (*GS*). ^a^Standard error of prediction reliability for the SMGS set was 0.02; ^b^standard error of prediction reliability for the SIRE set ranged from 0.06 to 0.08; ^c^standard error of prediction reliability for the SMGS set ranged from 0.03 to 0.05

### Individual SNP effects

**Fig. 2:**
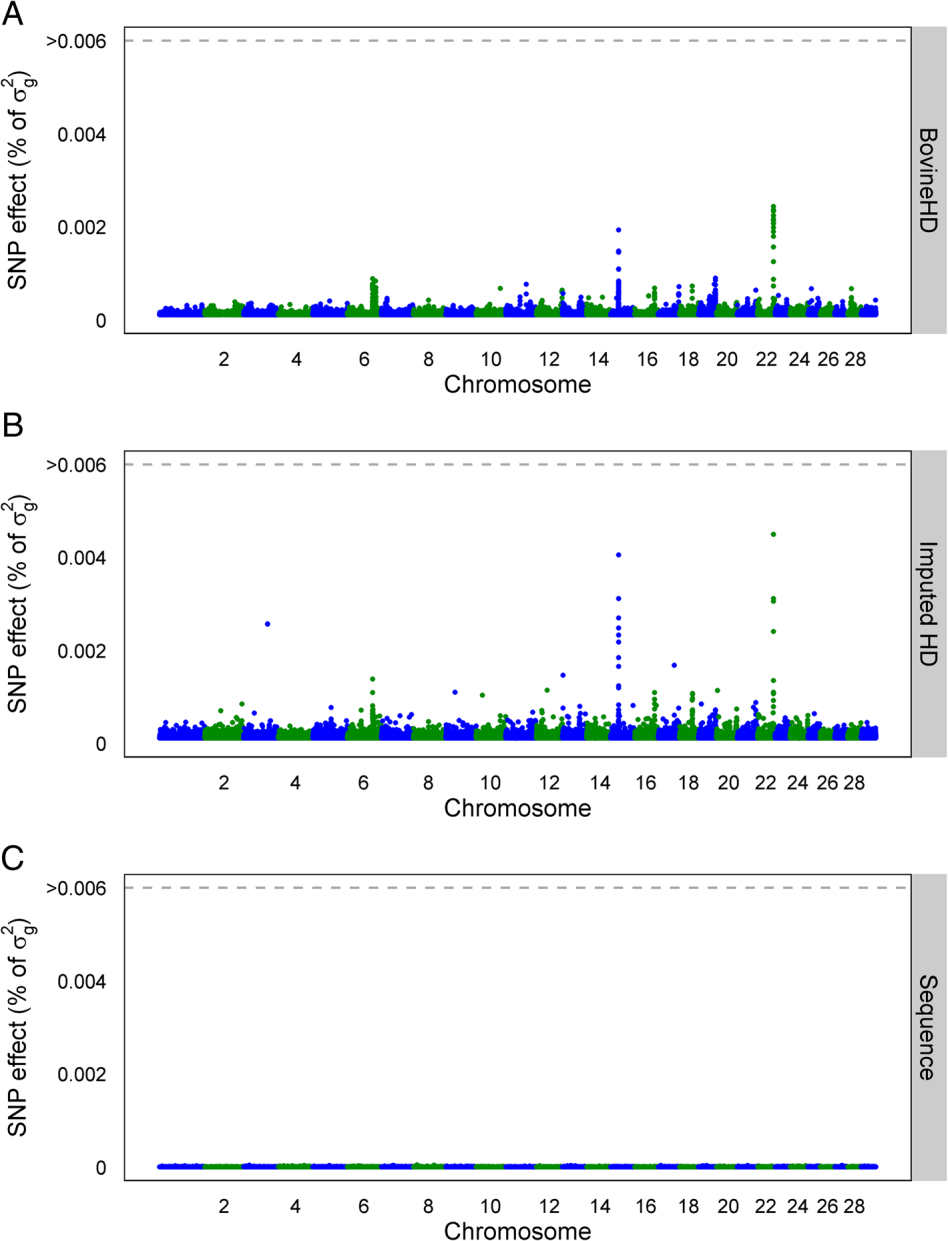
Manhattan plot with estimated SNP effects (% of *σ*_*g*_^2^) for somatic cell score (SCS) using the BSSVS model. Estimated SNP effects (% of *σ*_*g*_^2^) based on the BSSVS model for somatic cell score using BovineHD data (**a**), ImputedHD data (**b**), and imputed sequence data (**c**)

**Fig. 3:**
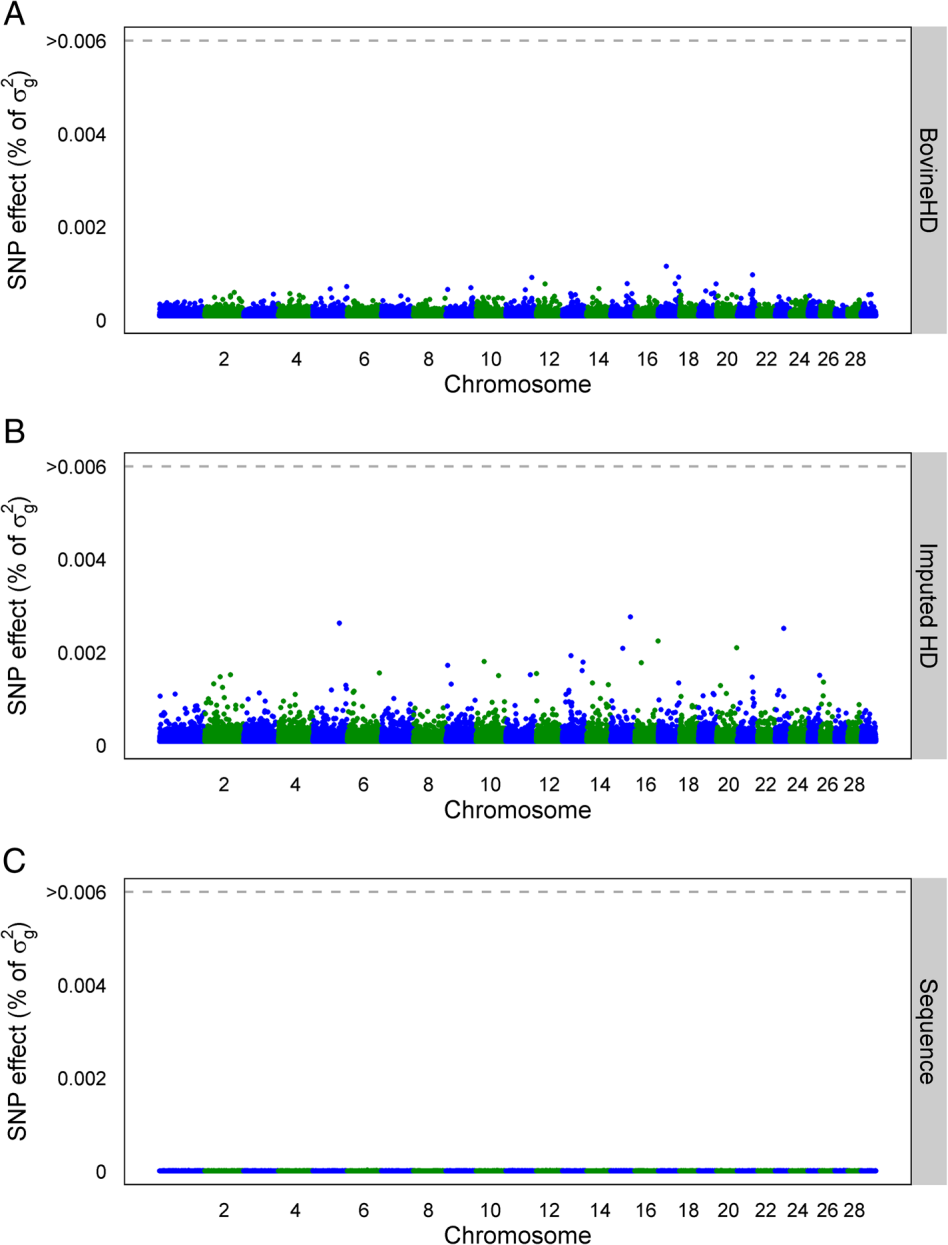
Manhattan plot with estimated SNP effects (% of *σ*_*g*_^2^) for interval between first and last lactation (IFL) using the BSSVS model. Estimated SNP effects (% of *σ*_*g*_^2^) based on the BSSVS model for interval between first and last lactation using BovineHD data (**a**), ImputedHD data (**b**), and imputed sequence data (**c**)

**Fig. 4:**
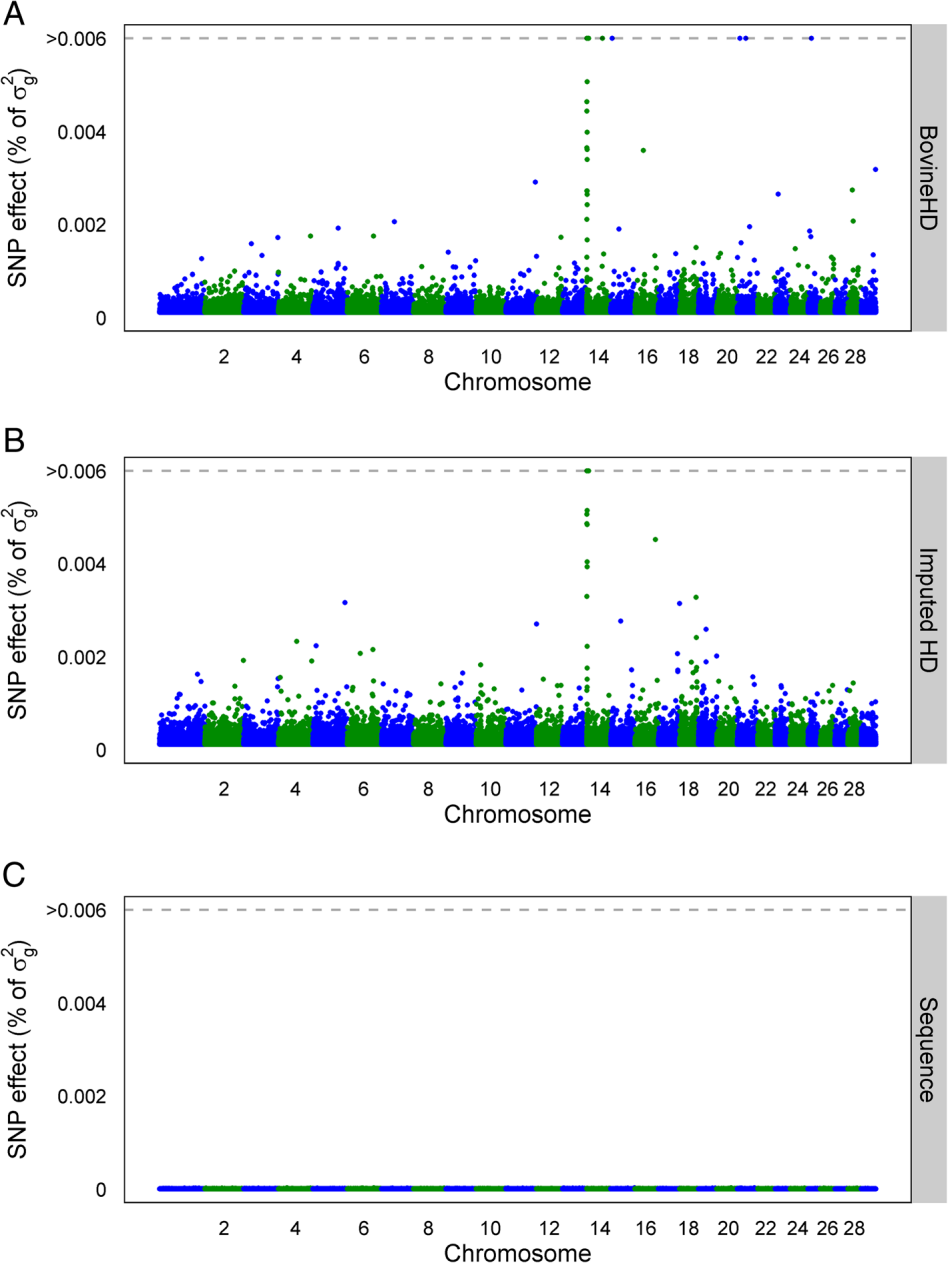
Manhattan plot with estimated SNP effects (% of *σ*_*g*_^2^) for protein yield (PY) using the BSSVS model. Estimated SNP effects (% of *σ*_*g*_^2^) based on the BSSVS model for protein yield using BovineHD data (**a**), ImputedHD data (**b**), and imputed sequence data (**c**)

For both genomic prediction methods, the (persistency in) reliabilities were highest when BovineHD genotype data were used compared to imputed sequence data. However, the additive genetic variances explained when imputed sequence data or BovineHD data was used were similar (Table 1). In Figs. 2, 3 and 4, the individual SNP effects are plotted (as % of *σ*_*g*_^2^) for BSSVS using BovineHD data, ImputedHD data, and imputed sequence data. These Manhattan-plots do not show similar genome-wide association results as typically seen from single-SNP analyses. Instead, the Manhattan-plots represent the variances explained by a single SNP, conditional on fitting all other SNPs simultaneously. Therefore, SNP effects are much smaller than those obtained when only one SNP is fitted. Still, the figures show that when BovineHD data and ImputedHD data are used for SCS and PY, it is possible to detect some regions on the genome that explain greater levels of variance, e.g. on chromosomes 15 and 22 (SCS) and chromosome 14 (PY). For BovineHD data, 26 SNPs had a SNP variance greater than 0.003 %, with a maximum of 0.05 %, most of these SNPs were located in a 1.8 Mb region at the beginning of chromosome 14. With imputed sequence data, no clear region could be detected with large SNP effects on the traits, but it should be noted that with imputed sequence data, there are 20 times more SNPs. For a fair comparison with BovineHD data, SNPs in the imputed sequence data were grouped in windows of 20 neighboring SNPs and the sum of the variances of the neighboring SNPs per window was plotted. However, still we did not detect any clear regions with an increased level of explained variance (results not shown).

## Discussion

Our objective was to investigate the reliability of genomic prediction based on imputed whole-genome sequence genotypes versus high-density SNP genotypes using real cattle data. Our hypothesis was that the use of sequence data in genomic prediction would result in higher reliability and higher persistency of reliability across generations. The rationale was that sequence data include the causal mutations that underlie QTL and that the effects of these mutations are estimated directly and not via effects of associated SNPs. This has been shown using simulated data [11–13] but not yet with real data. Contrary to our expectation, the results did not show a higher (persistency of) reliability of genomic prediction using imputed sequence data compared to BovineHD SNP genotypes, although a relatively large training dataset with highly accurate phenotypes based on many daughters was used. While we did not expect a large gain in prediction reliability, we did expect to see a small gain as has been reported in studies comparing genomic prediction using 50,000 and 777,000 SNPs [8–10]. Moreover, studies that used simulated whole-genome sequence data have claimed an increase in reliability using sequence data, e.g. [11]. The main improvement we expected was for persistency of reliability, when comparing the reliability observed in the lower related validation subset (GS) compared to more closely related validation sets. However, no increase in persistency of reliability was observed with imputed sequence data compared to the BovineHD data. In this study, our approaches were close to those used for genomic prediction in dairy cattle, including a training set of closely related animals, a pre-imputation step, and standard genomic prediction methods. Apparently, these approaches are not optimal to capitalize on the potential provided by sequence data. Below, we will discuss several factors that can explain this result.

### Dataset

Our results did not show an advantage of using imputed sequence data compared to BovineHD genotype data for genomic prediction. Using imputed sequence data, both genomic prediction methods missed some QTL or QTL regions e.g. (Figs. 2, 3 and 4). A reason for this could be the structure of the dataset. Animals in the training set used in this study were closely related to each other. For example, the training set included 2878 father-son relationships. Close relationships between animals cause long range LD between a SNP and the QTL. Long range LD is useful for genomic prediction of animals that are closely related to those of the training population. However, when the aim is to find the precise location of QTL based on sequence data, long-range LD between the training animals is unfavorable, for instance to increase accuracy of genomic prediction across generations or populations. In a simulation study using dairy cattle data, it was concluded that using a training set with animals that have a low average relationship is beneficial for genomic prediction [7]. Altogether, a training set with less related individuals (e.g. multiple breeds) might be required to increase the advantage of using sequence data for genomic prediction. However, because of the way breeding programs operate currently and because relationships contribute significantly to prediction accuracy, in practice, it may not be possible to avoid this problem, other than by using training populations that include multiple breeds or lines.

In this study, 3416 individuals were used to estimate the effects of over 12 million SNPs. Thus, the number of SNPs (*p*) was much larger than the number of observations (*n*), which might be a second limitation of the current training set. With a dataset that is too small, the QTL effects might be estimated with too much error, which reduces the advantage of using sequence data compared to SNP genotypes for genomic prediction [14]. The Manhattan plots in Figs. 2, 3 and 4 suggest that the effect of the potential QTL was spread across multiple SNPs. Increasing the number of individuals in the training dataset or pre-selecting SNPs based on other sources of information [36] might be necessary to increase prediction reliability based on sequence data, as reported by Hayes et al. [37]. These authors obtained a very small increase of 2 % in prediction reliability using imputed sequence data compared to BovineHD. However, they applied strict a-priori filtering steps for the SNPs and ended up with around 1.7 million variants, which is a factor 7 less than in our study. Also, their training set consisted of 16,214 bulls and cows, compared to the 3416 bulls used here. Thus, to benefit from the advantage of using sequence data compared to BovineHD genotype data for genomic prediction, it is necessary to aim for a large training set with a small average relationship between the animals, and possibly to pre-select SNPs based on functional information.

### Pre-imputation step

Apart from the size and structure of the training dataset, the quality of the pre-imputation step could also impact the advantage of using sequence data for genomic prediction. To really benefit from imputed whole-genome sequence data compared to BovineHD data, imputation accuracy should be greater than the LD (measured as r^2^) between a BovineHD SNP and the QTL. To test the possible effect of imputation, genomic prediction using a dataset of randomly selected SNPs from the imputed sequence data (ImputedHD) was compared with genomic prediction using the BovineHD dataset. Depending on the trait and method, a reduction of 0.01 to 0.03 in prediction reliability was found. A reduction in the reliability of GEBV with imputed genotypes has also been reported for studies on dairy cattle that used imputation from a few hundred SNPs to 50k SNPs, e.g. [38–42], which showed that the magnitude of the imputation errors was larger and the reliability of genomic prediction was lower compared to imputation from a 3k or 6k panel to a 50k panel. It has also been shown that the influence of imputation errors depends on the trait studied, e.g. traits that are influenced by a few large QTL were more affected than traits that are influenced by many QTL [38]. Moreover, van Binsbergen et al. [16] reported that the accuracy of imputation from BovineHD to sequence data ranged from 0.77 to 0.83 when the number of animals per breed ranged from 45 to 91. In this study, since 429 individuals from multiple breeds were used as reference animals, the accuracy of imputation was expected to be higher [16–18]. Although the accuracy of imputation was relatively high, imputation errors will have some effect. However, based on the results with the ImputedHD data, we believe that the errors in the pre-imputation step were probably a small factor in the reduction of the advantage of using sequence data compared to BovineHD data for genomic prediction.

The reason why imputation can reduce the accuracy of prediction is that imputed genotypes are called with increased uncertainty. In this study, SNPs that were likely to be imputed incorrectly were removed from the genotype dataset, using a low threshold of 0.05 for estimated imputation reliability to minimize the risk of removing potential causal mutations. With such a low threshold, there is still uncertainty about the genotype calling of imputed SNPs and potential causal mutations, although the mean imputation reliability was equal to 0.77. To take the effect of uncertainty in genotype calling on imputation accuracy into account, we considered the possibility of using the genotype probability instead of the most likely genotype for genomic prediction, which is expected to increase the reliability of genomic prediction [40]. However, using genotype probabilities, saved as real or double precision values, would increase computation requirements by a factor 4 or even 8 compared to using the integer values (0, 1, and 2) used in our study. With the currently available resources, using genotype probabilities was not feasible.

### Genomic prediction methods

A third reason why imputed sequence information did not improve prediction reliability could be parameterization of the BSSVS model. In the BSSVS model used here, we assumed that the prior distribution for *α*_*j*_ depended on the variance *σ*_*α*_^2^ and the QTL indicator *I*_*j*_, which was sampled for each SNP taking a value of 0 if the SNP was included in the model with a small $\left(\frac{\sigma_{\alpha}^2}{100}\right)$ effect or 1 if the SNP was included with a large effect (*σ*_*α*_^2^). With imputed sequence data, each cycle included about 12 million SNPs with a small effect. Combined together, these small SNP effects might explain a very large part of the variance and, thus, the larger QTL remained undetected by the model. A way to decrease the variance explained by the SNPs with a small effect could be to include only SNPs with large effects and set all other SNP effects to zero as:

$$ {\alpha}_j\Big|\pi, {\sigma}_{\alpha}^2=\left\{\begin{array}{c}\hfill 0\kern4.25em  when\ {I}_j=0\hfill \\ {}\hfill \sim N\left(0,{\sigma}_{\alpha}^2\right)\  when\ {I}_j=1.\hfill \end{array}\right. $$

This model is also known as BayesC [21]. Compared to BSSVS, BayesC will save computing time, since, in each cycle, for a large proportion of the SNPs, part of the calculations can be skipped as soon as *I*_*j*_ is sampled to be 0. Also, instead of two distributions, with large and (close to) zero effects, it might be useful to derive SNP effects from more distributions, which is done in methods such as BayesR [8].

It was assumed that both genotype panels had the same number of underlying QTL, i.e. the chosen *π* was larger for the imputed sequence dataset compared to the BovineHD dataset. However, due to LD between closely linked SNPs, the number of SNPs with a large effect might be larger for imputed sequence data than for the BovineHD data. Therefore, it might be better to use the same *π* for analyses using imputed sequence data as that for BovineHD analyses. Ultimately, the combination of the chosen *π* value and the parameterization of the model defines a priori the distribution of the effects [43], and thereby controls the posterior distribution of the effects. For instance, a study based on a 50k genotype dataset showed that the maximum SNP variances achieved with BSSVS with a *π* value of 0.999 were up to ten times as large as those achieved with BayesC with a *π* value of 0.9 [44]. To overcome this, *π* could be treated as unknown [21].

Due to the computation requirements of genomic prediction applied to imputed sequence data, it was unrealistic to test many different settings and models. For example, with the BSSVS model, one chain of 80,000 cycles took approximately 85 days on a High Performance Linux cluster containing Intel(R) Xeon(R) CPU E5-2660 with a clock speed of 2.20 GHz. GBLUP was less time demanding (~6 h), but required ~600 GB of RAM to store the genotypes. Due to efficient storing of the genotypes in the right-hand-side algorithm [23], the BSSVS model required less memory (~32 GB of RAM). These large computer requirements prevent fine tuning of the models used, but, at the same time, empirical studies have shown only small differences in prediction accuracy between available linear models [19]. The size of the training set used and the relationships between the individuals are probably more important factors than the choice of the model [19]. Therefore, it might be more beneficial to focus on the properties of the training set, than to test many different settings and models.

With 12 million SNPs, convergence of the Gibbs sampler can be rather low. Convergence of the BSSVS model was visually inspected by plotting the total SNP variance for each cycle of the Gibbs sampler (See Additional file 1). The pattern of the estimated SNP variance components across the cycles appeared to be quite stable. For a simple check, EBV were also calculated after 40,000 cycles and 60,000 cycles. For the three traits analyzed here, the correlation between these EBV and the final EBV after 80,000 cycles was higher than 0.999 (results not shown). Based on these assessments, we believe that the model did converge and that the potential impact of Monte Carlo errors was probably small.

It should be noted that in contrast to the GBLUP model, the BSSVS model includes pedigree data and uses a spike-slab prior for the SNP-effects, i.e. priors are mixtures of two densities: one with small variance (the spike) and one with large variance (the slab). The GBLUP model was based on equally weighted markers and did not include the pedigree separately. Therefore, the comparison between BSSVS and GBLUP involves not only two different models but also two different input sets and this could make interpretation of the results difficult. However, we tested the GBLUP model by including a polygenic component for SCS using the three types of genotype data (See Additional file 3: Table S2). Due to the high correlation between the pedigree-based relationship matrix and genomic relationship matrix, the model had difficulties to converge. Including a polygenic component gave less residual error variance and therefore a slightly higher heritability. In addition to a higher heritability, the model also introduced more bias in predictions. However, prediction reliabilities were similar to those obtained with the GBLUP model without a polygenic component. Due to the convergence issues and similar prediction reliabilities, the GBLUP model without a polygenic component was used in this study.

### SNP pre-selection

As shown in Table 1, predictions using imputed sequence data had similar additive genetic variance as predictions using the BovineHD data but, at the same time, the Manhattan plots using the sequence data in Figs. 2, 3 and 4 did not reveal any regions with large effects. This suggests that the effect of the potential QTL was spread across multiple SNPs that were in high LD with the QTL. A way to overcome this problem is to pre-select SNPs based on annotation information or their putative regulatory role [37, 45]. Incorporation of this biological information has shown potential for the detection of QTL [45] but did not result in higher reliability of genomic prediction [37]. Improving the accuracy of this biological information might improve detection of QTL and also increase the prediction reliability [37].

To test if reliability of genomic prediction increased by giving certain SNPs a higher prior, we included some SNPs as fixed effects in the GBLUP model. For SCS, the three SNPs (on chromosomes 6, 15, and 22) that explained the most variance in the BovineHD analysis (Fig. 2) were selected. For PY, a SNP in *DGAT1* (*diacylglycerol O*-*acyltransferase 1*) (Chr14:1802266) was selected, since *DGAT1* is known to have a major effect on milk production traits in Holstein Friesian cattle [46, 47]. For SCS, the prediction reliability did not change. However, for PY the prediction reliability increased from 0.47 to 0.51 for the BovineHD data and from 0.44 to 0.49 for the imputed sequence data. This suggests that pre-selecting SNPs and treating them as fixed effects or giving them a high prior might improve prediction reliability. However, this will be true only for SNPs that have a substantially large effect on the trait, such as *DGAT1*.

## Conclusions

Our results did not show an advantage of using imputed sequence data compared to BovineHD genotype data for genomic prediction. To investigate whether using (imputed) sequence data compared to BovineHD genotype data can be an advantage for genomic prediction, the use of a large set of animals with small average relationships, along with other properties of the training set used, should be considered. Genomic prediction models that incorporate biological information of the SNPs, or use a stricter SNP pre-selection procedure, might also increase the advantage of using (imputed) sequence data for genomic prediction.

## Additional files

Additional file 1:
**Conditional posterior densities BSSVS model.** This file describes the conditional posterior densities for the BSSVS model. (PDF 82 kb) Additional file 2: Figure S1. SNP variance components across cycles for the BSSVS model. Values are shown for somatic cell score (SCS), interval between first and last insemination (IFL), and protein yield (PY) for the three replicates using BovineHD data and imputed sequence data. **Figure S2.** Original versus predicted breeding values for somatic cell score for the two methods (GBLUP and BSSVS) using the three types of data (BovineHD, ImputedHD, and imputed sequence) for the 2087 validation animals. **Figure S3.** Original versus predicted breeding values for interval between first and last insemination for the two methods (GBLUP and BSSVS) using the three types of data (BovineHD, ImputedHD, and imputed sequence) for the 2087 validation animals. **Figure S4.** Original versus predicted breeding values for protein yield for the two methods (GBLUP and BSSVS) using the three types of data (BovineHD, ImputedHD, and imputed sequence) for the 2087 validation animals. (PDF 529 kb) Additional file 3: Table S1. Correlations between predictions of the different models. **Table S2.** Estimates of genetic parameters for GBLUP model including polygenic component for somatic cell score. (PDF 45 kb)
