# Supplementary material for: Genomic prediction using imputed whole-genome sequence data in Holstein Friesian cattle
Source: Genet Sel Evol. 2015 Sep 17;47(1):71. doi: 10.1186/s12711-015-0149-x (PMC4574568; doi:10.1186/s12711-015-0149-x)
Supplement: Additional file 1: — Conditional posterior densities BSSVS model. This file describes the conditional posterior densities for the BSSVS model. (PDF 82 kb) [file 12711_2015_149_MOESM1_ESM.pdf]

Additional file 1

Conditional posterior densities BSSVS model

The conditional posterior density of α_j is:

$$N\left(\hat{\alpha}_j;\ \frac{\omega_j\hat{\sigma}_e^2}{\mathbf{x}_j'\boldsymbol{D}^{-1}\mathbf{x}_j+\lambda_j}\right)$$

where $\hat{\alpha}_j$ is the conditional mean of the allele substitution effect at locus j, $\lambda_j=\frac{\omega_j\hat{\sigma}_e^2}{\hat{\sigma}_\alpha^2}$, where $\omega_j=1$ (if $I_j=1$) or $\omega_j=100$ (if $I_j=0$). The conditional posterior density of σ_α^2 was: $\sigma_\alpha^2|\alpha\sim\chi^{-2}(\nu_\alpha+n, S_\alpha^2+\boldsymbol{\omega}'\hat{\boldsymbol{\alpha}}^2)$, where $\hat{\boldsymbol{\alpha}}^2$ is a vector of squares of the current estimates of the allele substitution effects of all loci, that is weighted by vector $\boldsymbol{\omega}$. The conditional posterior distribution of I_j was:

$$\Pr(I_j=1)=\frac{\mathrm{f}(r_j|I_j=1)(1-\pi)}{\mathrm{f}(r_j|I_j=0)\pi+\mathrm{f}(r_j|I_j=1)(1-\pi)}$$

where $r_j=\mathbf{x}_j'\mathbf{D}^{-1}\mathbf{y}^*+\mathbf{x}_j'\mathbf{D}^{-1}\mathbf{x}_j\hat{\alpha}_j$ where $\mathbf{y}^*$ are the conditional DRPs (i.e. for each SNP $\mathbf{y}$ minus the sum of the estimated SNP effects of the other SNPs), and $f(r_j|I_j=\delta)$ is the probability density function, giving the probability that $I_j=0(\delta=0)$ or $I_j=1(\delta=1)$, and is proportional to $\frac{1}{\sqrt{v}}\mathrm{e}^{-\frac{r_j^2}{2v}}$, where $v=\left(\mathbf{x}_j'\mathbf{D}^{-1}\mathbf{x}_j\right)^2\frac{\sigma_{\alpha j}^2}{\omega_j}+\mathbf{x}_j'\mathbf{D}^{-1}\mathbf{x}_j\sigma_e^2$. The conditional posterior density of σ_u^2 and σ_e^2 were inverse-χ^2 distributions, respectively $\sigma_u^2|u\sim\chi^{-2}(m-2,\mathbf{u}'\mathbf{A}^{-1}\mathbf{u})$ and $\sigma_e^2|e\sim\chi^{-2}(m-2,\mathbf{e}'\mathbf{D}^{-1}\mathbf{e})$, where m is the number of animals in the pedigree.
