# Supplementary material for: Genomic prediction using imputed whole-genome sequence data in Holstein Friesian cattle
Source: Genet Sel Evol. 2015 Sep 17;47(1):71. doi: 10.1186/s12711-015-0149-x (PMC4574568; doi:10.1186/s12711-015-0149-x)
Supplement: Additional file 2: Figure S1. — SNP variance components across cycles for the BSSVS model. Values are shown for somatic cell score (SCS), interval between first and last insemination (IFL), and protein yield (PY) for the three replicates using BovineHD data and imputed sequence data. Figure S2. Original versus predicted breeding values for somatic cell score for the two methods (GBLUP and BSSVS) using the three types of data (BovineHD, ImputedHD, and imputed sequence) for the 2087 validation animals. Figure S3. Original versus predicted breeding values for interval between first and last insemination for the two methods (GBLUP and BSSVS) using the three types of data (BovineHD, ImputedHD, and imputed sequence) for the 2087 validation animals. Figure S4. Original versus predicted breeding values for protein yield for the two methods (GBLUP and BSSVS) using the three types of data (BovineHD, ImputedHD, and imputed sequence) for the 2087 validation animals. (PDF 529 kb) [file 12711_2015_149_MOESM2_ESM.pdf]

Additional file 2

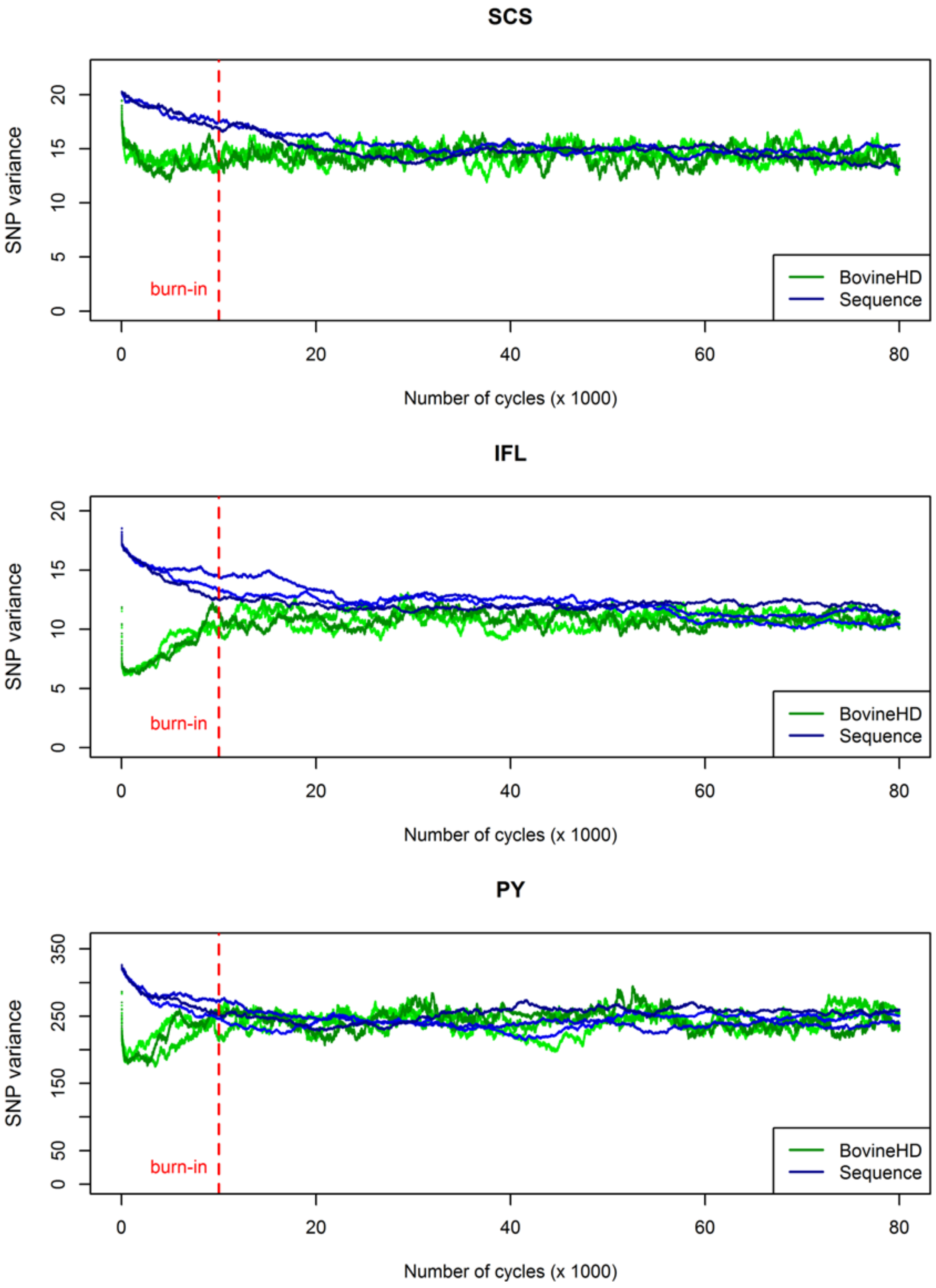

Figure S1 – SNP variance components across cycles for the BSSVS model
Values are shown for somatic cell score (SCS), interval between first and last insemination (IFL), and protein yield (PY) for the three replicates using BovineHD data and imputed sequence data.

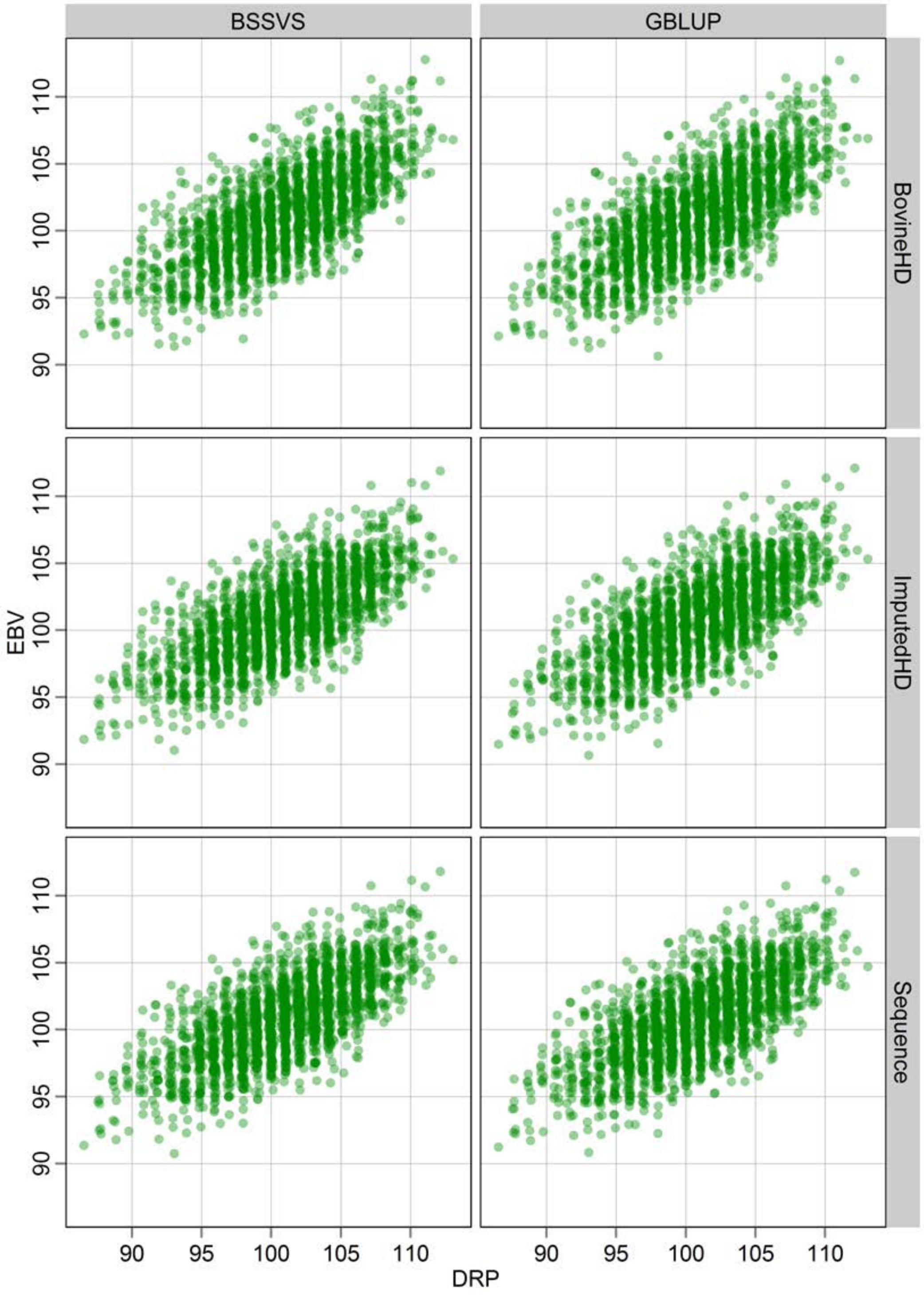

Figure S2 – Original versus predicted breeding values for somatic cell score
Original de-regressed proofs (DRP) versus the estimated genomic breeding values (EBV) for the two methods (GBLUP and BSSVS) using the three types of data (BovineHD, ImputedHD, and imputed sequence) for the 2087 validation animals.

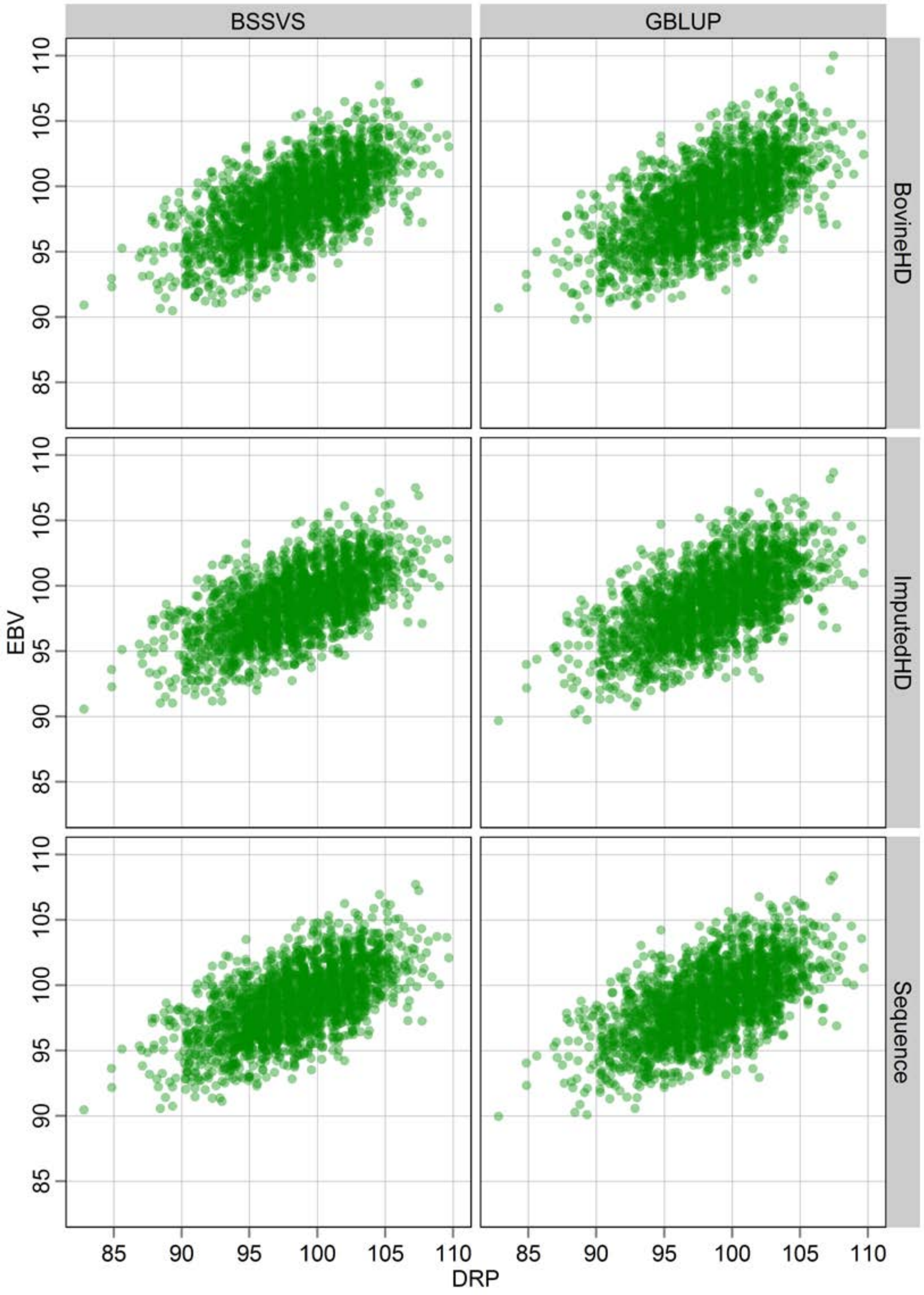

Figure S3 – Original versus predicted breeding values for interval between first and last insemination

Original de-regressed proofs (DRP) versus the estimated genomic breeding values (EBV) for the two methods (GBLUP and BSSVS) using the three types of data (BovineHD, ImputedHD, and imputed sequence) for the 2087 validation animals.

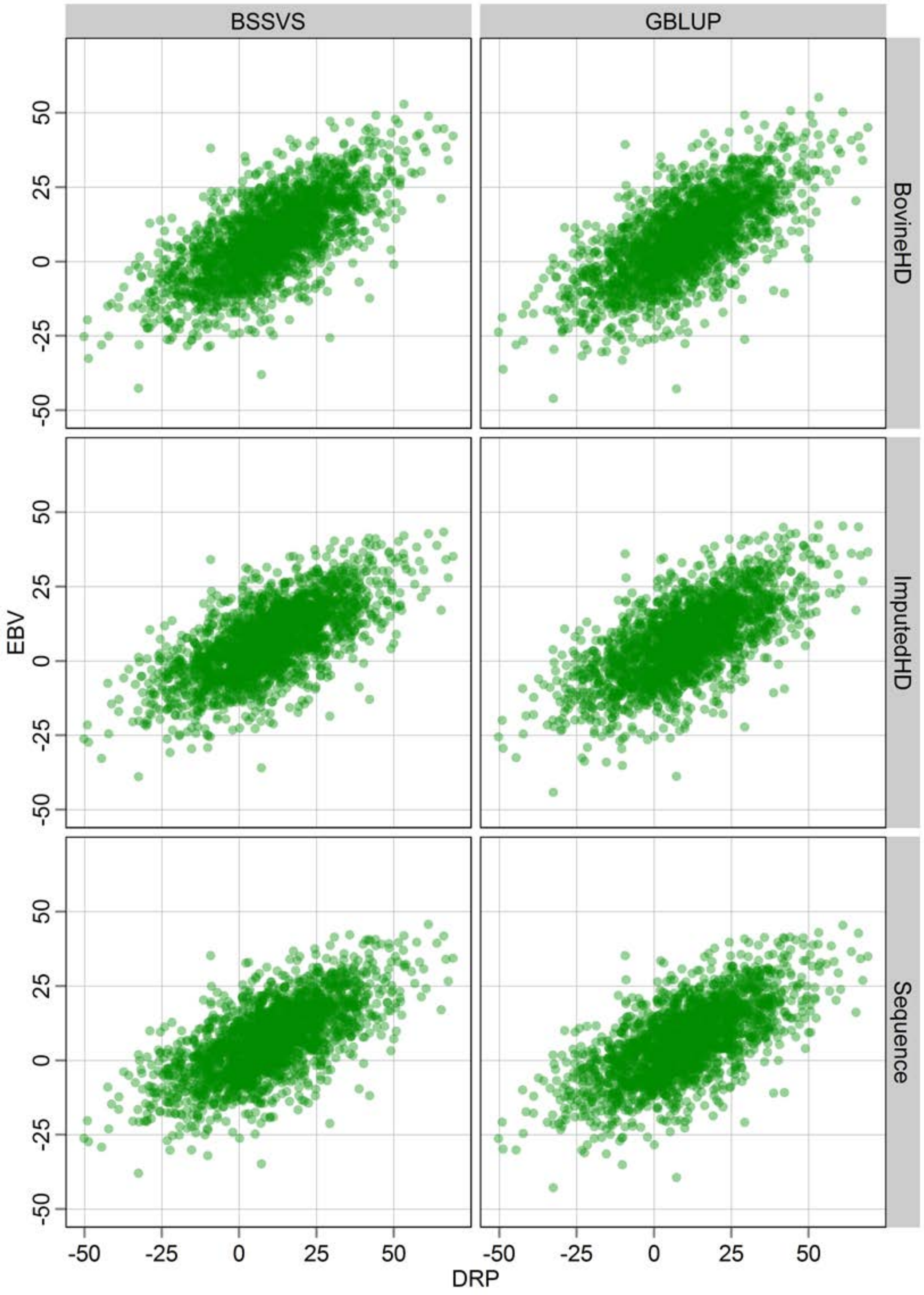

Figure S4 – Original versus predicted breeding values for protein yield

Original de-regressed proofs (DRP) versus the estimated genomic breeding values (EBV) for the two methods (GBLUP and BSSVS) using the three types of data (BovineHD, ImputedHD, and imputed sequence) for the 2087 validation animals.
