# Supplementary material for: Genomic prediction using imputed whole-genome sequence data in Holstein Friesian cattle
Source: Genet Sel Evol. 2015 Sep 17;47(1):71. doi: 10.1186/s12711-015-0149-x (PMC4574568; doi:10.1186/s12711-015-0149-x)
Supplement: Additional file 3: Table S1. — Correlations between predictions of the different models. Table S2. Estimates of genetic parameters for GBLUP model including polygenic component for somatic cell score. (PDF 45 kb) [file 12711_2015_149_MOESM3_ESM.pdf]

Additional file 3

Table S1 – Correlations between predictions of the different models

		Pedigree - BLUP	BovineHD - GBLUP	ImputedHD - GBLUP	Sequence - GBLUP	BovineHD - BSSVS	ImputedHD - BSSVS
SCS	BovineHD - GBLUP	0.751					
	ImputedHD - GBLUP	0.721	0.975				
	Sequence - GBLUP	0.720	0.973	0.996			
	BovineHD - BSSVS	0.792	0.995	0.969	0.967		
	ImputedHD - BSSVS	0.765	0.973	0.993	0.993	0.976	
	Sequence - BSSVS	0.765	0.974	0.992	0.995	0.977	0.998
IFL	BovineHD - GBLUP	0.700					
	ImputedHD - GBLUP	0.678	0.967				
	Sequence - GBLUP	0.680	0.967	0.996			
	BovineHD - BSSVS	0.795	0.978	0.948	0.949		
	ImputedHD - BSSVS	0.796	0.954	0.972	0.973	0.977	
	Sequence - BSSVS	0.771	0.961	0.979	0.983	0.977	0.997
PY	BovineHD - GBLUP	0.729					
	ImputedHD - GBLUP	0.717	0.965				
	Sequence - GBLUP	0.715	0.964	0.996			
	BovineHD - BSSVS	0.766	0.992	0.959	0.959		
	ImputedHD - BSSVS	0.758	0.959	0.988	0.989	0.968	
	Sequence - BSSVS	0.762	0.964	0.991	0.995	0.967	0.994

Table S2 – Estimates of genetic parameters for GBLUP model including polygenic component

Estimates of additive genetic variance (σ_g^2), heritability (h^2), regression coefficient (b), and prediction reliability (r^2) for somatic cell score using three types of genomic data.

Data	$\boldsymbol{\sigma_g^2}$	$\boldsymbol{h^2}$	$\boldsymbol{b}$ [(1)]	$\boldsymbol{r^2}$ [(2)]
**BovineHD**	18.44	0.95	1.12	0.51
**ImputedHD**	19.00	0.99	1.14	0.49
**Sequence**	18.82	0.98	1.16	0.49

[1] Standard error of the regression coefficient ranged between 0.02 and 0.03

[2] Standard error of the prediction reliability was 0.02.
